# Supplementary material for: Evolution of an Iron-Detoxifying Protein: Eukaryotic and Rickettsia Frataxins Contain a Conserved Site Which Is Not Present in Their Bacterial Homologues
Source: Int J Mol Sci. 2022 Oct 29;23(21):13151. doi: 10.3390/ijms232113151 (PMC9658677; doi:10.3390/ijms232113151)
Supplement: Supplementary file 1 [file ijms-23-13151-s001.zip › Supplemental Figure S1.pdf]

Supplemental Figure S1

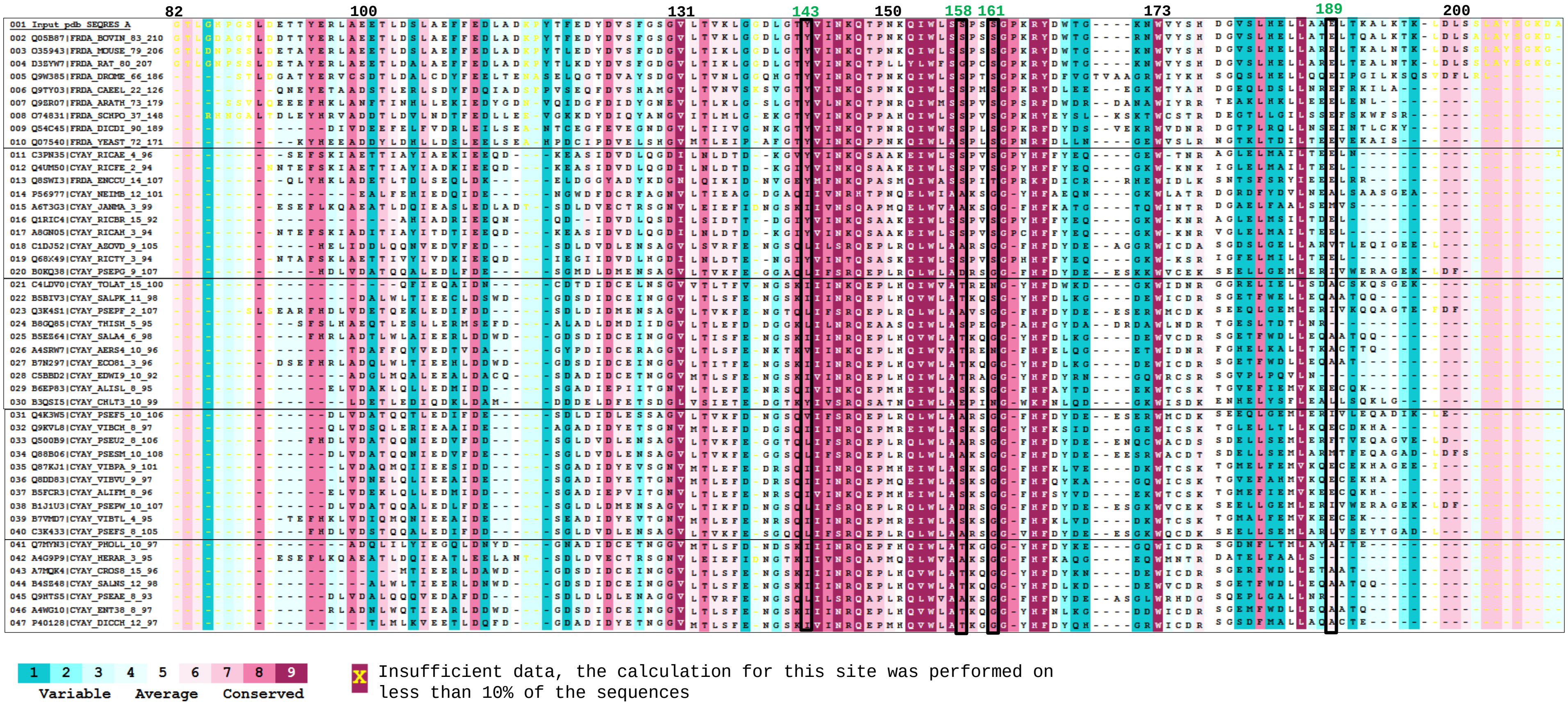

Supplemental Figure 1 - Multiple sequence alignment of Frataxin sequences used by ConSurf for analysis ConSurf-ID20. Residues are colored according to the conservation scores calculated by ConSurf. The input pdb sequence corrresponds to the sequence of human mature frataxin extracted from pdb entry 3S4M. Residues from conservation cluster 3 are boxed, and its position in the human frataxin sequence indicated by a green number.
