# Supplementary material for: Evolution of an Iron-Detoxifying Protein: Eukaryotic and Rickettsia Frataxins Contain a Conserved Site Which Is Not Present in Their Bacterial Homologues
Source: Int J Mol Sci. 2022 Oct 29;23(21):13151. doi: 10.3390/ijms232113151 (PMC9658677; doi:10.3390/ijms232113151)
Supplement: Supplementary file 1 [file ijms-23-13151-s001.zip › Supplemental Figure S2.pdf]

Supplemental figure S2

A

```
                                E111
94  TYERLAEETLDSLAEFFEDLADKPY  118 HUMAN
72  TYERVCSDTLDALCDYFEELTENAS   96 DROME
72  KYHEEADDYLDHLLDSLEELSEAHP   96 YEAST
    .*.. ..: ** * : :*:*::
```

B

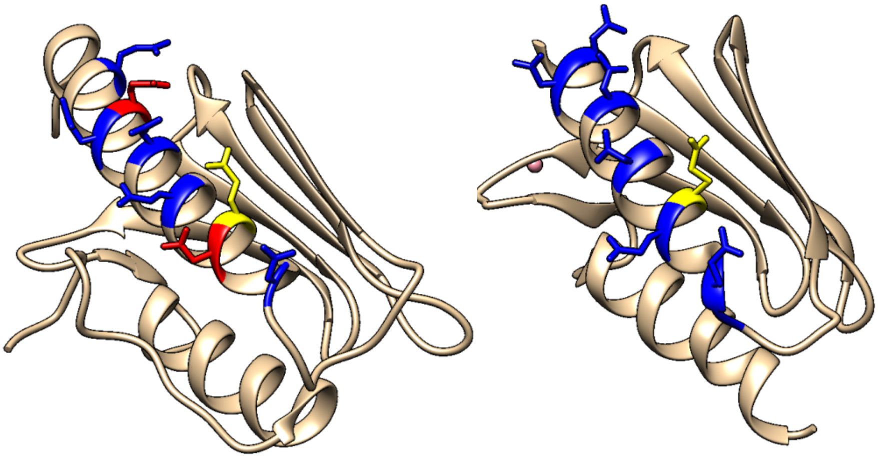

Supplemental Figure 2 - The frataxin acidic ridge. A, alignment of the sequence from the N-terminal helix alpha from three eukaryotic frataxins which present a group of acidic amino acids forming an acidic ridge. Acidic residues are highlighted, and the highly conserved E111 residue is boxed. B, Left, human frataxin (PDB ID:3S4M), right, S. cerevisiae frataxin (PDB ID:3OER). Acidic aminoacids are shown in stick representation. E111, identified as a highly conserved aminoacid is shown in yellow. Residues presenting variation in human GenomAD database (E100 and D112) are shown in red. The remaining acidic residues are shown in blue.
